# Study protocol. A prospective cohort study of unselected primiparous women: the pregnancy outcome prediction study

**DOI:** 10.1186/1471-2393-8-51

**Published:** 2008-11-19

**Authors:** Dharmintra Pasupathy, Alison Dacey, Emma Cook, D Stephen Charnock-Jones, Ian R White, Gordon CS Smith

**Affiliations:** 1 Department of Obstetrics and Gynaecology, University of Cambridge, Cambridge, UK; 2 MRC Biostatistics Unit, Institute of Public Health, Cambridge, UK

## Abstract

**Background:**

There have been dramatic changes in the approach to screening for aneuploidy over the last 20 years. However, the approach to screening for other complications of pregnancy such as intra-uterine growth restriction, pre-eclampsia and stillbirth remains largely unchanged. Randomised controlled trials of routine application of high tech screening methods to the general population have generally failed to show improvement in outcome. We have previously reviewed this and concluded it was due, in large part, to poor performance of screening tests. Here, we report a study design where the primary aim is to generate clinically useful methods to screen women to assess their risk of adverse pregnancy outcome.

**Methods/design:**

We report the design of a prospective cohort study of unselected primiparous women recruited at the time of their first ultrasound scan. Participation involves serial phlebotomy and obstetric ultrasound at the dating ultrasound scan (typically 10–14 weeks), 20 weeks, 28 weeks and 36 weeks gestation. In addition, maternal demographic details are obtained; maternal and paternal height are measured and maternal weight is serially measured during the pregnancy; maternal, paternal and offspring DNA are collected; and, samples of placenta and membranes are collected at birth. Data will be analysed as a prospective cohort study, a case-cohort study, and a nested case-control study.

**Discussion:**

The study is expected to provide a resource for the identification of novel biomarkers for adverse pregnancy outcome and to evaluate the performance of biomarkers and serial ultrasonography in providing clinically useful prediction of risk.

## Background

The current pattern of provision of antenatal care for low risk women in the UK was established in 1929. Major changes have taken place in certain aspects of care, in particular, population-based screening for fetal abnormality [1]. These have been driven by technological developments in ultrasound, biochemical screening and molecular genetics. In contrast, the methods for screening the low risk population for other complications of pregnancy, in particular, intra-uterine growth restriction, pre-eclampsia and stillbirth, have remained largely unchanged over recent years [2]. The normal approach is to assess women for risk factors in their medical, gynaecological and obstetric history at the booking visit. This is followed by serial antenatal visits with a midwife which include measurement of the symphysis-fundal height with a tape measure, measurement of blood pressure with a sphygmomanometer, and urinalysis. The recent guideline from the UK National Institute for Clinical Excellence (NICE) on antenatal care addresses the assessment of "Fetal growth and well-being in unselected women" (section 1.10). The sole positive statement is that "Symphysis-fundal height should be measured and recorded at each antenatal appointment from 24 weeks" [3]. However, routine measurement of symphysis-fundal height had a sensitivity of less than 30% for detection of small for gestational age (SGA) infants, even though the relatively broad definition of birth weight less than the 10^th ^percentile for gestational age was employed [4].

One of the primary reasons for assessing fetal growth and well-being is to prevent stillbirth, defined as death of an infant in the perinatal period showing no signs of life. Stillbirth accounts for 60% of all perinatal deaths and 75% of all potentially preventable losses (defined as perinatal death of a normally formed infant weighing 1000 g or more) [5]. Analysis of the determinants of stillbirth leads to the conclusion that over half are related to placental dysfunction and that many of these are associated with impaired fetal growth [2]. Placental function can be assessed by utero-placental Doppler flow velocimetry and fetal growth can be assessed by ultrasonic fetal biometry. It would seem plausible that routine application of these tests may prevent stillbirth. However, meta-analysis of randomised controlled trials demonstrates that use of neither methodology reduced perinatal mortality when applied to unselected women [6,7]. Hence, in the UK and many other countries, it is not recommended that these tests are routinely offered to pregnant women. We have previously reviewed the literature around stillbirth and concluded "future work on population-based screening for stillbirth should be preceded by high-quality, non-interventional prospective cohort studies characterising the screening properties of new methods of risk assessment in an unselected population" [2]. Here we describe a protocol for such a study focusing on assessing and developing biochemical and ultrasonic indicators of placentation.

## Methods/design

The study design is a prospective cohort study. The cohort is defined as primiparous women with a singleton pregnancy who are attending for their dating scan at the hospital's ultrasound department. The initial conduct of this study is confined to a single centre (The Rosie Hospital, Cambridge, UK). However, it has the potential to be conducted at other centres.

### Recruitment and first appointment

Woman with a positive pregnancy test are told by their midwife to contact the ultrasound department to arrange a dating scan. When women contact the department, they are asked if they have had any previous pregnancies progress beyond 23 weeks. If they have not, a letter of invitation and a patient information leaflet for the study are sent. Women are approached following their dating scan. Hence those with non-viable or multiple pregnancies are not approached for consent. Consent is obtained by a research midwife. Participation is explained, any questions are answered and women who agree sign the consent form. The original is retained by the study, a copy is given to the participant and a copy is placed in her clinical case record. Routine antenatal care at the hospital involves an ultrasound scan at 20 weeks for a detailed anatomical scan of the fetus. For women who consent to the project, this appointment is made on a research list where the appointment is of one hour in duration (routine duration is 30 minutes). Appointments (30 minute) for research scans at 28 and 36 weeks are also made at this time and, finally, the participant is sent for phlebotomy.

### Second appointment

At the second appointment, each participant has their detailed scan of fetal anatomy. Any abnormalities or complications are dealt with by standard hospital protocols. At the time of the scan, further information is obtained, including maximum thickness of the placenta, the presence and dimensions of lakes, and umbilical and uterine artery Doppler flow velocimetry. The results of the research component of the scan are not reported. At this visit, a brief computer assisted questionnaire is conducted. The elements of this are tabulated (Table 1). Data from the first scan are also retrieved and all information is stored directly onto a custom built database linked to a web-based interface and designed using Microsoft SQL and ASP website. Maternal height and weight are also measured and phlebotomy performed. Pre-pregnancy weight is self reported.

**Table 1:** Maternal demographic data

**Characteristics**
Name
Date of birth (dd/mm/yy)
Current age (yr)
Marital status (married/cohabitating/single)
Occupation (free text)
Partner's occupation (free text).
Discontinued full time education (Y/N)
Age full time education stopped (yr)
Smoking status
(never/quit pre-pregnancy/quit during pregnancy/currently smoking)
If currently smoking, number per day
Alcohol use (units per week)
Current
Pre-pregnancy
Current medical condition (free text)
Current prescription medication (free text)
Previous pregnancies ending less than 24-weeks (Y/N)
If yes 1. Gestational age (wks) 2. Spontaneous (Y/N)
Use of oral contraceptive pill in the last 3 months (Y/N)
Date of last menstrual period (dd/mm/yy)
Certain of date last menstrual period (Y/N)
Duration of menstrual cycle (/28 days)

The study also involves collection of a sample for DNA from the partner and this is usually obtained at the second appointment as the partner is often present for this scan. An information leaflet was offered for the woman's partner at the first visit. Where the partner attends, the research sonographer explains participation, answers any questions and obtains written consent to obtain a sample of saliva using the Oragene^© ^DNA collection kit. The partner's height and weight are also measured.

### Third and fourth appointment

These visits are conducted at 28 and 36 weeks gestational age, respectively. The formats for both are identical. A scan is performed which includes fetal biometry, amniotic fluid index, uterine and umbilical uterine artery Doppler flow velocimetry, and assessment of placental maturity. In addition, maternal weight is measured and phlebotomy is performed. Most of the scan information is concealed. The software on the machines which generates gestational age estimates based on biometry is disabled preventing ad hoc estimation of the appropriateness of measurements. Four items of information are revealed if present, namely (1) previously unrecognised major congenital abnormality, (2) previously unrecognised placenta praevia, (3) profound oligohydramnios (amniotic fluid index less than 5) and (4) presentation at 36 weeks.

### Phlebotomy and serum/plasma storage

Samples are taken into a plain tube and an EDTA tube for serum and plasma, respectively. They are spun in a centrifuge at 4°C, each sample is then separated into four aliquots and placed in a -40°C freezer. Twice a day samples are removed from the freezer and stored at -80°C. All are split into pairs and stored in separate freezers. All freezers are equipped with identical racking systems. The samples are placed in 1.8 ml tubes, with 81 tubes in a box. A total of 28 boxes are placed in a rack and the freezer contains 18 racks. Hence, each freezer has the capacity to store 40,824 tubes. The location and identity of all samples is stored using a sample inventory software package, Pro-Curo (Brady Laboratories), and samples are identified using a bar coded label. Samples for maternal DNA are stored as whole blood. The paternal salivary sample is processed and paternal DNA is also stored at -80°C. All freezers are connected to a secure power supply, supported by the hospital's emergency generator, which is regularly tested. All freezers are also equipped with a liquid CO_2 _back-up system (New Brunswick Scientific) which will maintain samples at less than -55°C for 20 hours in the event of failure of a freezer. All freezers are connected to a phone line and will send an automated message to technical staff in the event of a failure or a rise of temperature greater than -65°C. A third empty freezer is maintained at -80°C in order to accommodate samples in the event of failure of one of the main freezers.

### Delivery

**Figure 1:**
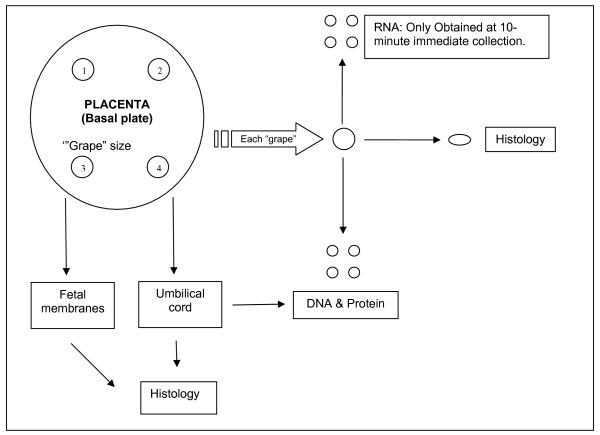
Schematic of placental collection at delivery.

Pregnant women receiving antenatal care in the UK National Health Service generally carry hand held notes. Women participating in the study have their hand held notes contained in a plastic folder with the study logo to flag their participation. Moreover, a sticker is placed in the delivery unit section of the notes to flag the need for placental collection and a sheet with instructions is included in the notes. This sheet includes the number for a phone held by the on-call technician. If a woman delivers at a time when a technician is present in the hospital, the placenta is passed to the technician as soon after delivery as practicable. The placenta is sampled as described in the schematic shown in Figure 1. Details of the collection protocol for the samples to be used for histological, DNA, RNA and protein analyses are given in Table 2. If the woman delivers at a time when there is no technician present, the placenta is placed in a dedicated fridge and the samples processed the following morning. The details again are described in Table 2.

**Table 2:** Placental Collection

**Immediate level Collection**	
Completed within 10 minutes of delivery of the placenta	
1. Placenta	Sites at the periphery of four lobules, free of visible infarction, calcification, haematoma or damage are identified. 1–2 mm of tissue is removed from the maternal surface (basal plate) and discarded. A "grape size" sample of placental tissue is then obtained from each site and washed repeatedly in chilled phosphate buffered saline. The following samples are obtained from each 'grape':
	i. RNA: Four 5 mg pieces placed in RNA later, (Applied Biosystems, Warrington, UK), and stored at -80°C.
	ii. DNA & protein: Four 50 mg pieces, frozen in liquid nitrogen and stored at -80°C.
	iii. Histology: A "pea-sized" sample is fixed in 3 ml of formalin (24 hours at 4°C) and embedded in paraffin wax.
2. Placental membranes	A 2 × 2 cm portion of the membranes is obtained and fixed in formalin as above.
3. Cord blood	A 10 ml anti-coagulated (EDTA/citrate) sample is obtained. Peripheral blood mononuclear cells are isolated and stored in liquid nitrogen.
4. Umbilical Cord	The following samples are obtained
	i. DNA & protein: Two 100 mg pieces of cord, frozen at -80°C.
	ii. Histology: One 5 mm transverse section of cord is fixed in formalin as above.

**Basic level collection**	
Placenta is stored in a fridge and sampled within 24 hours of delivery.	

1. Placenta	Four 'grape size' samples are obtained and washed as described above. From each 'grape' sample, the following are obtained:
	i. DNA & protein: Four 50 mg pieces, frozen at -80°C.
	ii. Histology: A "pea-sized" sample is fixed in 3 ml of formalin as above.
2. Placental membranes	A 2 × 2 cm portion of the membranes is obtained and fixed in formalin as above.
3. Umbilical cord	The following samples are obtained
	i. DNA & protein: Two 100 mg pieces of cord, frozen at -80°C.
	ii. Histology: One 5 mm transverse section of cord is fixed in formalin as above.

### Ethics

The study was approved by the Cambridge Local Research Ethics Committee 2. Ethics applications in the UK include a section, "A68. What are the main ethical issues with the research?" The issue flagged in this section was our decision to conceal most of the information retrieved in the research scans, with the exceptions listed above. The committee raised no concern regarding this arrangement but did request changes in the nature and presentation of the information in the patient information leaflets.

### Ascertainment of outcome

All outcomes will be ascertained by trained midwives performing direct examination of the woman's clinical record. Standardised definitions will be employed, such as the ACOG classification of pre-eclampsia [8]. In addition, the research database will be linked using the case record number to the hospital's IT systems. This will allow retrieval of routine blood investigations, other targeted ultrasonic investigations, and the Delivery Unit record. Details will also be obtained regarding admission of the infant to the neonatal intensive care or special care units.

### Analytic approach

The study design allows three major analytic approaches, namely, analysis as a cohort study, a case-cohort study or a nested case-control study. All three approaches are planned. Analysis as a cohort study will employ the information retrieved on all participants. This includes all the maternal demographic information, information on the partner, the mother's height and weight serially measured through the pregnancy, and the result of the research ultrasound scans. Analysis as a case-cohort design will be used to determine the associations and predictive ability of biochemical and genetic measurements on the stored samples. In this approach, a random sample of the cohort (the sub-cohort) is selected as the control group at the start of the study. Biochemical and genetic measurements are made on the sub-cohort and on all cases. The analysis for a given adverse event compares cases with the members of the sub-cohort who did not experience the given outcome. The advantages of the case-cohort study design are that a common group of controls can be selected for all adverse outcomes [9]. It has the advantage over simply performing the given biochemical assay on all cohort members of substantially reducing the costs of analysis with minimal loss of precision.

Case-cohort studies with time-to-event outcomes require non-standard statistical analyses but those with binary outcomes do not [10]. Thus statistical analysis of both cohort and case-cohort designs will be performed using logistic regression [11]. In order to avoid over-fitting of models, multivariate analyses will use a cross validation approach. A model will be fitted on 90% of the given study group and then the probability of the outcome calculated for each member of the remaining 10%. This process will be repeated until "out of sample" estimated probabilities are obtained for all participants in the given analysis. The screening performance of the models will be assessed using this predicted probability in two ways. First, the area under the receiver operating characteristic (ROC) curve will be estimated, with 95% confidence intervals as a summary of its discrimination. Different models will be compared using the method of DeLong et al [12]. Second, the screening performance of models will be assessed by analysis using percentiles of predicted probability, where women in the top 5%, 10% or 20% of predicted probability are regarded as screen positive. The screening performance of each of the thresholds will then be summarized in terms of sensitivity, specificity, positive and negative predictive values and likelihood ratios.

Finally, we also plan to conduct nested case-control studies within the cohort. This approach will be used where very expensive or labour intensive methodologies are planned, such as proteomic analysis of serum or gene expression array analysis of placental RNA, and will involve sampling of cases as well as controls. These methods are generally utilized in studies trying to understand the biology of adverse pregnancy outcome or to identify novel biomarkers. Nevertheless, the possibility of confounding is still present with these approaches. Multivariate analysis is impractical using these methods, given that small numbers are employed. Our approach is to perform a nested case-control design with one to one matching of cases and controls on key maternal characteristics to address the potential for confounding. Subsequent analyses will, therefore, utilise paired statistical methods.

### Power calculations

Clearly power calculations cannot be performed prospectively for all potential analyses in the study. Moreover, we aim to study a series of adverse outcomes which have different incidence rates. A key measure we aim to determine is the sensitivity of different models for a given screen positive rate. The precision of any estimate of sensitivity can be quantified by the 95% confidence intervals. If we take the example of a model with 80% sensitivity, the 95% CI will be 74–85% for 200 cases, 71–87% for 100 cases, 66–90% for 50 cases and 56–94% for 20 cases. Taking the example of a condition with 3% incidence (e.g., delivery of an infant with birth weight less than the 3^rd ^percentile), we would obtain 60 cases with a cohort of 2000 women and 120 cases with 4000 women. Hence, the study is likely to provide reasonably precise estimates of sensitivity for conditions with a 3% incidence. The incidence of preterm birth and pre-eclampsia are both likely to be slightly higher [13,14]. However, the incidence of some other complications, such as stillbirth, typically 0.5% in nullipara, will be lower [15]. Hence, we have an open ended plan for recruitment, as the study will progressively achieve power for the less common adverse outcomes with greater cohort size.

### Initial experience with study

This study commenced at the Rosie Hospital, Cambridge UK on the 14^th ^of January 2008. Over the first six months, a total of 588 eligible women were approached and 340 (57.8%) agreed to participate in the study and provided written consent. Over this period 4 (1.2%) women have withdrawn, two of whom have relocated. No woman has withdrawn due to any problems with the conduct of the study and no significant negative feedback has been given, formally or informally.

## Discussion

We describe a simple design for a prospective cohort study of unselected primiparous women. We will compare this study with two other large scale prospective cohort studies in pregnancy, namely, the Southampton Women's Survey [16] and SCOPE [17]. The Southampton Women's Survey (SWS), funded by the MRC in the UK, was a prospective cohort study which recruited women in their reproductive years and then aimed to study these women during their pregnancy. A total of 12,500 women were approached and approximately 3000 liveborn infants are to be studied through childhood. SCOPE is a multicentre prospective cohort study recruiting primiparous women at around 15 weeks gestational age. It commenced in Auckland, New Zealand, but now also has centres in Australia, the UK and Ireland and is still actively recruiting. Each study will have its strengths and drawbacks. We will briefly discuss the rationale for the approach we have taken.

In our study, we have limited recruitment to primiparous women. There were essentially four reasons for doing so. First, primiparity is associated with increased risks of a number of adverse outcomes of pregnancy, including preterm birth, pre-eclampsia, stillbirth and intrapartum caesarean section [18-20]. By focusing on primiparous women, there will be a greater proportion of the cohort who experience adverse events. Second, one of the best predictors of the outcome of pregnancy is the outcome of a woman's previous pregnancy. This information is clearly not available for primipara, hence, there is a particular need for tests to predict risk in this population. Third, any model derived from women of mixed parity would have to incorporate previous pregnancy outcome for those who were multiparous. In a cohort of mixed parity, this information will be variably present and hence complicate the process of modelling. Finally, we plan to run this study for at least 4 years. It is likely that a sub-set of women will attend the hospital for consecutive pregnancies. This would mean that, within the cohort of pregnancies, some would be to the same women. Non-independence of observations is a further complicating factor for statistical analysis.

One feature of the present study is that we aim to recruit women early in pregnancy. We have done this as there is considerable evidence that complications in late pregnancy may be determined to a large extent in the first trimester (see Smith 2004 [21] for review). Women in the SWS cohort were recruited pre-pregnancy. However, this was a major undertaking and the ratio of women approached to women who were followed through to pregnancy was greater than 4:1. While there are clearly real strengths in a study design where pre-pregnancy data are available, this involves the expense of retrieving information from large numbers of women who do not have a pregnancy in the timescale of the study. Understanding the relationship between a mother's diet prior to pregnancy and the subsequent health of her child was a key issue in the SWS but is not a focus of the present study, hence the different approach.

A important feature of the present study is that we perform serial scans and phlebotomy through all three trimesters of pregnancy. The SWS study performed scans at 19 and 34 weeks and the SCOPE study at 20 and 24 weeks. The rationale for first trimester assessment is developed above. The basis for scans and phlebotomy at 28 and 36 weeks, in addition to the early sampling, is previous analyses of other datasets. We have previously reported the association between circulating concentrations of alpha fetoprotein and human chorionic gonadotrophin in maternal blood at 15–20 weeks gestation and the risk of stillbirth [15]. Both measures were much better predictors of stillbirths at extreme preterm gestation than losses in later pregnancy. Similarly, high resistance patterns of uterine artery Doppler flow velocimetry at 23 weeks gestation are strongly predictive of stillbirth at extreme preterm gestations, but weakly associated with later losses [22]. The primary intervention to prevent stillbirth is elective delivery and this carries serious risks to the mother and infant if performed at extreme preterm gestations. This provides a rationale for improving methods of detecting babies at risk of stillbirth at later gestational ages (see review [2]). Hence, we have included ultrasonic and biochemical tests at later gestations.

A distinctive feature of the present study is that relatively basic information is retrieved about the mother. This is in contrast to the SWS where a 90 minute interview was conducted prior to the pregnancy and food frequency questionnaires and diaries were administered both in early and late pregnancy. It is also in contrast to the SCOPE study. This involves a 60–90 minute interview at 15 weeks and a 45 minute interview at 20 weeks. As other studies are addressing the factors that can only be ascertained through questionnaires, there is less impetus to have a further study addressing the same issues. Moreover, detailed questionnaires addressing environmental, dietary and social history are time consuming to administer. This reduces the number of women who can be recruited for a given number of personnel hours. It also may serve as a relative obstacle to recruitment if women feel inhibited about a prolonged and detailed interview. In order to optimise recruitment, the present study was designed to make involvement attractive to potential participants. The total time involved through participation in this study is less than two hours across all four visits. Moreover, more than half of that time is spent having ultrasound scans performed and women generally feel very positive about ultrasonography in pregnancy [23]. We interpret the greater than 50% recruitment rate and the very low drop out rate as confirming the attractiveness and acceptability of this study design. The attractiveness of participation is important as it may tend to encourage women from a wider range of social and educational backgrounds. A design which systematically recruits very highly motivated and educated women will result in reduced rates of adverse events and a highly selected population may also adversely affect a study's external validity. Finally, our study has at its core the aim of assessing markers of placentation in the prediction of pregnancy outcome. The study design reflects our belief that combined ultrasonic and biochemical assessment of the placenta is likely to be a better predictor of adverse outcome than maternal dietary or other history.

When designing the present study, a key question was whether information obtained through a research scan should be used for ad hoc screening of research participants. With the very few exceptions we allowed, the position in this study was that the research scan data should not be so employed. However, women have all ultrasonic investigations that are normally conducted and have any additional scans requested by the attending obstetrician, i.e. participating in this study does not alter in any way the routine or targeted scanning requested by clinical staff caring for those recruited. The basis for concealing the research scan information was recommendations from NICE in the UK. Their guideline on antenatal care, which is intended to inform all NHS activity in the UK, explicitly states that routine Doppler and growth studies should not be performed on unselected women [3]. The basis for their position is the result of meta-analyses of randomised controlled trials of these methods which failed to show any benefit [6,7]. We flagged this issue in our application for ethical approval and the study received approval. Other studies have, however, taken different views. The SWS study revealed the results of a growth scan at 34 weeks. The SCOPE study reveals the following information: cervical length <15 mm at 20 weeks, absent or reversed end diastolic velocity on umbilical artery Doppler at 24 weeks, and abdominal circumference <10^th ^percentile at 24 weeks.

The initial target recruitment for the present study is 4000 women. We estimate that this would yield approximately 120 cases of small for gestational age infants (using the strict definition of <3^rd ^percentile), 150–200 preterm births, and about 80 cases of significant pre-eclampsia (the number of cases of pre-eclampsia will depend on the stringency of the diagnosis). These should be adequate for most analyses planned. However, antepartum stillbirth unrelated to congenital abnormality affects about 0.5% of unselected primigravid women who attend for prenatal screening [15]. Hence, we would anticipate only approximately 20 such events. This is the basis for planning open-ended recruitment. However, samples from small numbers of women experiencing rare events will still be used to identify novel biomarkers using techniques such as proteomics and there may be potential to validate candidate markers in other cohorts. We will also use non-lethal complications of pregnancy, such as intra-uterine growth restriction, abruption and severe pre-eclampsia, as proxies for stillbirth. This is consistent with the view that similar processes operate in the lethal and non-lethal manifestations of these conditions [24].

The study is designed both to provide samples to evaluate blood tests as predictors of adverse pregnancy outcome and to provide very well phenotyped pregnancy outcome for basic science studies. In the former case, we will use multivariate modelling to address potential confounding. In the latter case, samples will be provided for basic science studies as matched cases and controls. Many translational research studies which employ an observational design essentially ignore the possibility of confounding. However, women who experience pregnancy complications exhibit multiple differences when compared to the healthy population. Moreover, medical interventions will tend to be used selectively. Taking an example of Western blot analysis of placental expression of a protein comparing women with and without pre-eclampsia, the cases and controls are likely to differ in relation to age, parity, obesity and smoking status [13,25]. Moreover, women with pre-eclampsia are more likely to be delivered preterm and more likely to be delivered by planned caesarean section. Many studies applying basic science methods to the study of human clinical material fail to account adequately for these systematic differences and many such analyses are conducted in a way that would be unacceptable in clinical epidemiological research. We will address this using a nested case-control design with one to one matching on key maternal characteristics. This approach reflects a key aim of this study i.e. to combine excellence in science with excellence in clinical research design and analysis.

In summary, we present a simple pragmatic design for a prospective cohort study of unselected primigravid women. This involves quite intensive scanning, phlebotomy and collection of other samples, but appears to be acceptable to the majority of such women in our initial experience.

## Competing interests

The authors declare that they have no competing interests.

## Authors' contributions

GCSS designed the study with input from DSCJ and IRW. GCSS, DP, IRW and DSCJ drafted the manuscript. DP, EC and AD produced the detailed working protocol. All authors read and approved the final manuscript.

## Pre-publication history

The pre-publication history for this paper can be accessed here:


